# Tele-medicine in respiratory diseases

**DOI:** 10.1186/s40248-017-0090-7

**Published:** 2017-04-20

**Authors:** Nicolino Ambrosino, Dewi Nurul Makhabah, Yusup Subagio Sutanto

**Affiliations:** 1 European Respiratory Society (ERS), Lausanne, Switzerland; 2 Pulmonary and Respiratory Medicine Department, Medical Faculty Sebelas Maret University, Solo, Central Jawa Indonesia

**Keywords:** Chronic respiratory failure, COPD, Ehealth, ICT, Neuromuscular diseases, Tele-monitoring, Tele-rehabilitation, Ventilator assisted individuals

## Abstract

Information and Communication Technologies applied to health care and advances in sensor and data transmission technology allowed tele-medicine based programs of care also for patients with respiratory diseases.

Different sensors, transmission devices and interventions are used in tele-medicine for some indications. Patients suffering from Chronic Obstructive Pulmonary Disease, asthma, neuromuscular diseases, ventilator assisted individuals and those undergoing pulmonary rehabilitation programs may benefit from this approach.

The legal problems are still unsolved. Economic advantages for health care systems, though potentially high, are still poorly investigated.

Despite the hopes, we need more evidence before this modality can be considered as a real progress in the management of patients with respiratory diseases. On one hand, these technologies can improve the care of patients with difficult access to services, particularly those in rural/remote areas, on the other hand, there is the risk that they will be used only to reduce standard services in health systems of developed countries.

## Background

The increased life expectancy of worldwide population [1] results, and will result even more in the next future, in high prevalence of chronic and non-communicable diseases, as well as of complex patients with “chronical criticities” also due to respiratory diseases. As a consequence, the health care systems of the industrialised countries will have to face high burden also in the attempt to fulfill somehow unrealistic citizens’ expectations in the age of welfare decline [2, 3]. On the other hand, also goverments of low- and lower-middle-income countries have to face the increasing health needs of their populations, often in rural/remote areas [4]. With the purpose of reducing health care related expenses and delivering health facilities as much as possible, a prospective solution might be to care patients at home with the help of wearable technologies.

## Main text

### Tele-medicine

Wide application of Information and Communication Technologies (ICT) to health care organisations and advances in sensor and data transmission technology have allowed the development of tele-medicine based programs of care [5]. Tele-medicine has been defined as “the distribution of health services in conditions where distance is a critical factor, by health care providers using ICT to exchange at distance information useful for diagnosis” [6]. We can go beyond this definition as tele-medicine might be useful also to improve the delivery of and patients’ compliance to chronic management [7].

### Devices

Many devices can be used [8]:

- *Biological sensors* of patient’ s vital signs and physiological data such as: spontaneous breathing tidal volume, respiratory and heart rate, pulsossimetry, capnography;
- Data from *medical equipment* such as tidal volume, pressures of mechanical ventilators;
- Devices for *transmission* of data from those sensors and equipments such as: phone calls, sms, email, video phones, websites or mobile phones, video-conferencing;
- medical *devices programmed* at distance;
- dedicated internet *softwares*.

### Interventions

Different interventions may use those devices [9, 10]:

- Real time or “store and forward” video or telephone links between patients and care-givers in both directions;
- Internet-based tele-communication;
- Digital/broadband/satellite/wireless or Bluetooth transmission of physiological parameters with feedback to the patient.

### Indications

Tele-medicine has been applied to conditions such as [6, 11–14]:

- chronic heart failure
- diabetes
- chronic obstructive pulmonary disease (COPD)
- chronic respiratory failure (CRF)
- neuromuscular diseases (NMD)
- tele-monitoring of ventilator-assisted individuals (VAIs)
- home based pulmonary rehabilitation (tele-rehabilitation)
- stroke
- behavioral health
- staff education and training
- primary care

### Chronic obstructive pulmonary disease

The effects of tele-medicine in COPD patients are still under discussion [15].

### Pro

Systematic reviews and meta-analyses have reported benefits [16]. A study showed that the quality of spirometry performed by non professionals improved by means of at distance collaboration between primary care professionals and lung function specialists [17]. Clinical benefits have been shown also in severe COPD patients with comorbidities [18]. In patients with CRF a tele-assistance program resulted in reduction in hospital admissions, General Practioner calls, and in costs [19]. A study in COPD patients with CRF on long-term oxygen, reported that a tele-medicine program alone and with greater efficacy when added to non invasive ventilation reduced the exacerbations rate [20]. Another small study indicated an association with reduction in hospital and emergency department admissions, and hospital length of stay [21].

### Con

Recent research did not confirm that these systems are more effective and less expensive than standard care [22]. In a 6 month crossover randomised controlled trial in patients with chronic respiratory diseases, addition of tele-monitoring to standard care did not improve the time to next hospitalisation or health related quality of life (HRQL), whereas it increased hospital admissions and home visits [23]. A systematic review did not find any conclusive evidence for the effectiveness of telephone follow up alone or with other tools in reducing readmissions in patients with chronic diseases [24]. A recent systematic review reports that only three out of the eighteen studies fulfilling the criteria for inclusion, found significant improvements in HRQL with tele-medicine [25]. Furthermore, the suggestion that tele-medicine could encourage the COPD patients’ self-management was not confirmed [26].

### Asthma

Tele-health has been used to support self-management of long-term conditions such asthma. Positive results have been reported [9, 27, 28]. A systematic review and meta-analysis from three randomised controlled trials using different technologies showed an improvement of asthma control, though the clinical effectiveness of the used apps, typically incorporating multiple features, varied [29].

### Neuromuscular diseases

The feasibility of tele-assistance for NMD patients with impaired cough capacity was assessed in a pilot study [30]. Patients’ respiratory signs and symptoms were recorded at home and transmitted to a remote control center and chest physiotherapy was prescribed and modulated accordingly. This modality was associated with reduced hospitalisations and emergency room admissions [30]. Use of tele-medicine is relevant in those NMD patients under home mechanical ventilation (HMV) [6].

### Tele-monitoring of ventilator dependent individuals

Approximately 13 to 20 million patients worldwide require life support in intensive care units (ICU) each year [31]. Advances in management have improved their mortality and morbidity. As a consequence the prevalence of VAIs is increasing in people with CRF due to advanced diseases such as COPD, restrictive thoracic diseases, and NMD [32]. The last reported, and underestimated prevalence of European patients requiring HMV is 6.6 per 100,000 population [33]. More recent Canadian data report a 12.9 prevalence [34], whereas another survey reports 9.9 and 12.0 prevalences in Australia and New Zealand, respectively [35]. The prevalence of HMV in Catalonia, Spain is reported to be 23 per 100,000 [36]. These patients have poor outcomes, despite high medical resource consumption [37]. The need to reduce health care costs and to improve safety has developed tele-monitoring programs for VAIs. A European Respiratory Society (ERS) Task Force produced a statement on accepted indications, follow-up strategies, equipments, facilities, legal and economic issues of tele-monitoring of these patients [6]. Variable models of care exist for VAIs [38]: a tele-monitoring program might be a key element in HMV organisation but it is difficult to assess without considering it in the frame of the comprehensive management of these patients in each country.

### Tele-rehabilitation

**Fig. 1:**
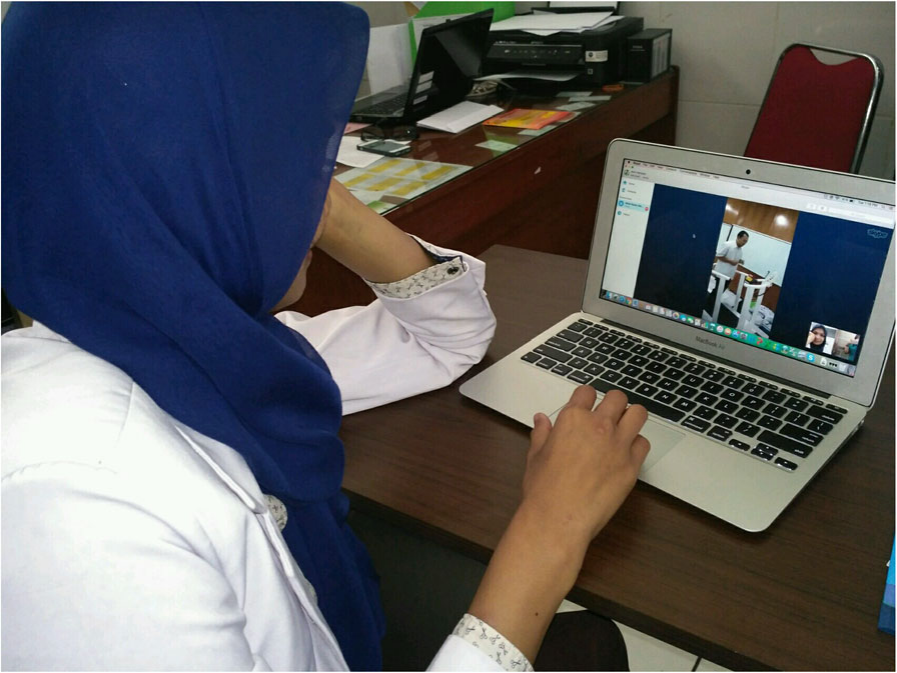
Patient exercising under supervision at distance

Pulmonary rehabilitation is suggested for the vast majority of COPD patients [39]. As a consequence to fulfill all needs, health care systems should face relevant organisational problems and resources consumption. Tele-rehabilitation might offer a valid aid. Tele-rehabilitation uses different models of services [11, 12, 30]. Patients may perform exercises at home under supervision by a physiotherapist who may prescribe and change strategies and settings at distance (Fig. 1). It has been shown that supervised home training and counseling patients, may be associated with safety, feasibility and benefits for severe COPD patients [40]. Home-based maintenance tele-rehabilitation was found to be equally effective to hospital-based, outpatient, maintenance pulmonary rehabilitation, in reducing acute exacerbations and hospitalizations and the risk for emergency department visits [12]. Nevertheless, another study, compared with the standard rehabilitation, did not find any significant improvement in COPD patients equipped with a tablet after 7–10 weeks of rehabilitation [41].

### Legal issues

Any application of tele-medicine must be considered a medical act, therefore there are legal problems with telemedicine still lacking shared international and national solutions. Therefore, users must use precautions [6, 42] in order to avoid problems such as:

- At distance consultation between patient/family and staff or among health operators may not reach appropriate standard of care;
- equipment or system may fail with ominous consequences [43];
- electronic data may be poorly protected and manipulated;
- distinction of responsibilities and potential obligations among each care-giver may be difficult.

With the large diffusion of this technology, law cases will increase, therefore, National and EU governments should promote common, ethical, legal, regulatory, technical, and administrative standards [44].

### Economic considerations

The economical impact of tele-medicine has been evaluated in a meta-analysis [45] indicating a decrease in hospitalization costs and additional savings. A systematic review concluded that synchronous or real time video communication were cost-effective for local delivery of services between hospitals and primary care [46]. Nevertheless, in current literature reports of costs are inconsistent and often obtained from studies of poor quality. As a consequence decision-makers may have difficulties in introducing this service in health systems. However, to evaluate the real cost/effectiveness of any new method of care such as tele-medicine, the definition of “standard therapy” in each study must be specified in the frame of the different home care organisations of each country [44].

### Problems

Patients’ age, education, experience in technological devices, cognitive, motor and visual abilities or deficits, phonation and speech abilities, their families and home environment, play an important role in the use of technologies of tele-medicine programs. The training to such technologies and programs should be directed to care-givers and patients in order to make them able to act in accordance with predefined protocols. Major obstacles limit the wider diffusion of tele-medicine as shown in Table 1. These barriers must be solved [47].

**Table 1:** Obstacles to the development of tele-medicine [5, 14]

• Lack of knowledge among patients, citizens and even professional care-givers.	
• Lack of connections among different systems.	
• Lack of clear evidence of cost-effectiveness.	
• Fears of potential legal conflicts.	
• Lack of transparency on data utilisation.	
• Reimbursement issues.	
• High initial costs.	

## Conclusions

While seeking savings through these new perspectives, health care organisations should not forget the quality of care. Tele-medicine should not be considered only to save resources at the price of reduction in quality and safety. These technologies can improve the care of patients with difficult access to services, particularly those in rural/remote areas [48] like in Indonesia, a country with more than 17,000 islands and with one of the highest prevalence of smoking habit. On the other hand, this approach might be an alibi to reduce standard services in more developed health systems. Despite the hopes in tele-medicine as a means of patients care, we need much more evidence before this modality can be considered as a real improvement in the management of patients.
